# Complete Nucleotide Sequence of the Mitogenome of *Tapinoma ibericum* (Hymenoptera: Formicidae: Dolichoderinae), Gene Organization and Phylogenetics Implications for the Dolichoderinae Subfamily

**DOI:** 10.3390/genes13081325

**Published:** 2022-07-25

**Authors:** Areli Ruiz-Mena, Pablo Mora, Eugenia E. Montiel, Teresa Palomeque, Pedro Lorite

**Affiliations:** Department of Experimental Biology, Genetics Area, University of Jaén, Paraje las Lagunillas s/n, 23071 Jaen, Spain; armena@ujaen.es (A.R.-M.); pmora@ujaen.es (P.M.); emontiel@ujaen.es (E.E.M.); tpalome@ujaen.es (T.P.)

**Keywords:** ants, mitogenome evolution, ant phylogeny, mitochondrial rearrangements

## Abstract

The ant *Tapinoma ibericum* Santschi, 1925 is native to the Iberian Peninsula. This species, as well as other species from the *Tapinoma nigerrimum* complex, could form supercolonies that make these species potentially invasive and could give rise to pests. Recently a mature colony from this species has been found in the Isle of Wight (United Kingdom). Mitogenomes have been used to study the taxonomy, biogeography and genetics of species, improving the development of strategies against pest invasion. However, the number of available mitogenomes from the subfamily Dolichoderinae is still scarce and only two of these mitogenomes belong to *Tapinoma* species. Herein, the complete mitogenome of *T. ibericum* is presented in order to increase the molecular information of the genus. The *T. ibericum* mitogenome, retrieved by Next-Generation Sequencing data, is 15,715 bp in length. It contains the typical set of 13 protein-coding genes, 2 ribosomal RNA genes, 22 transfer RNAs and the A + T-rich control region. Comparisons of the *T. ibericum* mitogenome with other dolichoderine mitogenomes revealed the existence of four gene rearrangements in relation with the ancestral insect mitogenome. One of these rearrangements, involving the *tRNA*-*Ile*, *tRNA-Gln* and *tRNA-Met* genes, was found in most of the analyzed ant mitogenomes. Probably this rearrangement was an ancestral or plesiomorphic character in Formicidae. Interestingly, another rearrangement that affects to *tRNA-Trp*, *tRNA-Cys* and *tRNA-Tyr* genes was found only in *Tapinoma* species. This change could be a synapomorphic character for the genus *Tapinoma*, and could be used as a phylogenetic marker. Additionally, a phylogenetic analysis was performed using the protein-coding gene sequences from available Dolichoderinae mitogenomes, as well as mitogenomes from representative species from other Formicidae subfamilies. Results support the monophyletic nature of the genus *Tapinoma* placing it within the same clade as the rest of Dolichoderinae species.

## 1. Introduction

Ants are eusocial insects of the family Formicidae (Hymenoptera) which can play a fundamental role in ecological processes. Ants are ecosystem engineers that move and aerate large volumes of soil. They are major predators of small invertebrates, plant pollinators and seed dispersers, plant symbionts, etc. [1]. This successful insect group includes more than 14,000 described species [2], making up a 15–20% of total terrestrial animal biomass [3]. Some ant species are known to be highly invasive organisms that can expand their distribution worldwide, contributing to the global biodiversity crisis [4]. These species have become successfully established outside their native ranges and they may cause several economic and environmental damage in the invaded areas [5]. The genus *Tapinoma* includes one of the most common invasive species of ants in the world, *T. melanocephalum*, widely distributed across the Old Word and New World in both hemispheres [6]. *Tapinoma ibericum* is restricted to the Iberian Peninsula although recently a mature colony was found in the Isle of Wight (United Kingdom) [7]. Morphological and molecular analyses using a fragment of the mitochondrial *cox1* gene proved that *T. ibericum* belongs to the *Tapinoma nigerrimum* complex, together with *T. darioi*, *T. nigerrimum* and *T. magnum* [7]. *T. ibericum*, *T. magnum* and *T. darioi* are supercolonial and are potentially invasive species that could give rise to pests [7].

Mitochondrial sequences have been used to provide a good knowledge of the taxonomy, biogeography, and genetic diversity of native and introduced species of ants in order to improve the development of optimal strategies against their invasion [8,9]. Invasive species not only reduce the genetic variability of native species, but can also hybridize with them, which can also be a problem for the conservation of native species [10]. Interspecific hybridization and genetic introgression between introduced and native species could be analyzed using mitochondrial DNA. Because mitochondrial DNA is maternally inherited it can indicate the direction of hybridization [10]. Hybridization is a relatively common process in ants [11]. The analysis of mitochondrial *cox1* gene suggests hybridization and genetic introgression between species of the *Tapinoma nigerrimum* complex [7].

Next-Generation Sequencing (NGS) data have allowed the easy retrieval of complete mitogenomes due to their high ratio of copy number to nuclear DNA [12]. Before the NGS techniques became more popular and affordable, mitogenomes have been sequenced by PCR amplification of overlapping fragments; even today, missing fragments are still being amplified by PCR, such as the control region or some missing fragments [13,14]. 

Although phylogenies using complete mitogenomes are very useful, the number of available complete mitogenomes from species of the subfamily Dolichoderinae is still scarce. Most of these mitogenomes have been sequenced in the last five years, and include only two *Tapinoma* species [15,16]. In this paper, we present, for the first time, molecular information about the complete mitogenome of *T. ibericum*, retrieved by NGS data. The sequence and gene organization of this mitogenome have been compared with other Formicidae species. Phylogenetic comparisons with other ant species were also carried out. 

## 2. Materials and Methods

### 2.1. Sample Collection and DNA Extraction

Specimens of *T. ibericum* workers were collected from a nest in El Portichuelo, Jaén, Spain, (37.727 N, 3.803 W). *T. ibericum* is not an endangered or protected species thus we did not need any specific permission for its collection. The workers were preserved in absolute ethanol at −20 °C until the DNA extraction. Genomic DNA was isolated using the NucleoSpin Tissue kit (Machery-Nagel GmbG & Co., Düren, Germany) following the instructions provided by the fabricant.

### 2.2. Sequencing and Mitogenome Assembly 

Low-coverage sequencing was performed using the Illumina^®^ Hiseq™ 2000 platform, yielding about 2.6 Gb data of 151 bp pair-end reads at Macrogen (Japan Corp. Tokyo, Japan). Raw data was filtered by quality using Trimmomatic v.0.36 [17]. The mitogenome was assembled de novo using NOVOPlasty (v4.3.1) [18]. NOVOPlasty uses the NGS data to assemble organelles’ genomes and needs a seed that will be iteratively extended in both directions. For the assembly we have used the *cox1* gene from *T. ibericum*, retrieved by mapping the mitogenome of *Linepithema humile* with the Illumina PE reads using bbmap (available in sourceforge.net/projects/bbmap/, accessed on 31 March 2022) and UGENE [19]. Then, we created a consensus sequence based on the alignment, and used this sequence as a seed. This seed can be extended and used for initiating the assembly or it can be used to retrieve one sequence read of the targeted genome from the NGS dataset [18]. Here, the seed was used only to start the assembly. Another feature that can be set up is the addition of a reference genome to perform the analysis. The run was carried out using the *L. humile* (GenBank acc. number KX146468) mitogenome as a reference [20]. The last feature to be set was the K-mer length. In our run, we tested several lengths, with the length of 33 being the most suitable. 

### 2.3. Mitogenome Annotation and Sequence Analysis

The mitogenome of *T. ibericum* was annotated according the procedure described by Cameron [21] using the web-based services MITOS [22] (available in http://mitos.bioinf.uni-leipzig.de, accessed on 31 March 2022). The annotation of protein-coding genes (PCGs) was refined manually by checking for consistent start/stop codons, open reading frames and by comparison with other Dolichoderinae mitogenomes using Geneious v10.1.3. The base composition was estimated using the BioEdit program (v7.0.9.0) (http://www.mbio.ncsu.edu/BioEdit/bioedit.html, accessed on 31 March 2022), and codon usage was analyzed using MEGA X [23]. The circularized plot of the mitogenome was carried out using Geneious v10.1.3. The resulting assembly and annotations were deposited in GenBank under the accession number ON746721.

Available Dolichoderinae mitogenomes were recovered from GenBank. The dataset included 16 sequences belonging to 11 species (Table 1). Several sequences of the same species have been included because they have different origins and sizes. Four of the selected mitogenomes were not annotated and only the sequences were available (*D. lamellosus*, *D. pustulatus*, *L. erythrocephalus* and *T. sessile*). In order to make comparisons, the mitogenomes of these species were annotated in the same way used for *T. ibericum* (Supplementary Tables S1–S3). 

### 2.4. Phylogenetic Analysis

For phylogenetic analysis, all available Dolichoderinae mitogenomes were used. In addition, mitogenome sequences of representative species from other Formicidae subfamilies were also used: *Ooceraea biroi* (accesion no. CM010870), *Anoplolepis gracilipes* (MH122734), *Formica fusca* (LN607805), *Myrmica scabrinodis* (LN607806), *Solenopsis invicta* (HQ215538), *Cryptopone sauteri* (MK138572), *Brachyponera chinensis* (MT215089), *Tetraponera aethiops* (BK010476), *Pseudormyrmex gracilis* (BK010472). As an outgroup, *Apis mellifera mellifera* PCG (KY926884) from the family Apidae was used.

The concatenated PCGs were aligned using MAFFT v7.453 software [30]. Poorly aligned positions and divergent regions were removed using the Gblocks program v.0.91.1 [31] (available at https://ngphylogeny.fr/tools/tool/276/form, accessed on 31 March 2022). After trimming, the dataset was 9287 bp in length. Phylogenetic relationships were reconstructed using the Maximum Likehood (ML) method implemented in MEGA X [23] using the GTR + G + I model, the model with lowest BIC (Bayesian Information Criterion) value.

## 3. Results and Discussion

### 3.1. Gene Organization

**Figure 1:**
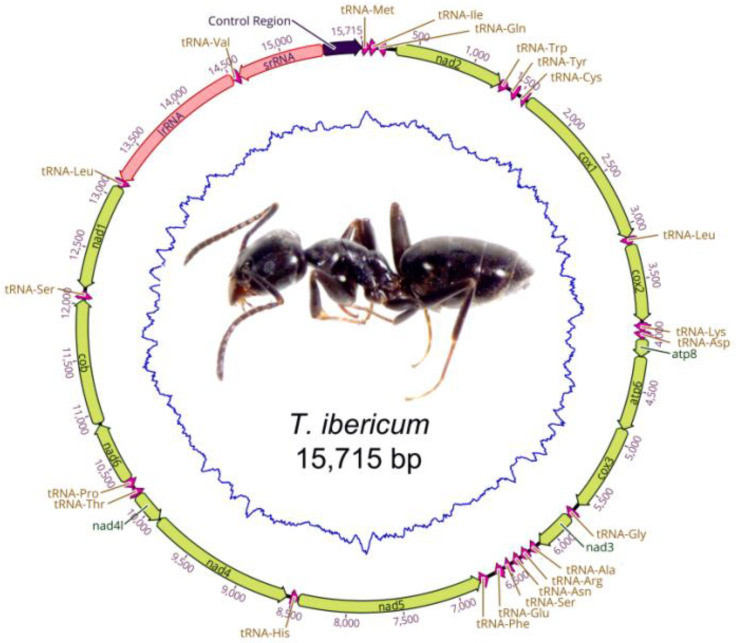
Graphical map of the mitogenome of *Tapinoma ibericum*.

The complete mitogenome of *T. ibericum* is 15,715 bp in length, close to the mitogenome sizes of *T. melanocephalum* (15,499 bp) or *T. sessile* (15,287 bp) and other sequenced Dolichoderinae mitogenomes, in which the mitogenome sizes ranged from 15,287 to 16,259 bp (Table 1). The *T. ibericum* mitogenome contains the typical set of 13 protein-coding genes (PCGs), 2 ribosomal RNA genes (rRNAs), 22 transfer RNAs (tRNAs) and the A + T-rich control region (Figure 1, Table 2). The size variations observed in Dolichoderinae mitogenomes were due to differences in the intragenic spacer sizes and mainly in the control region.

Four PCGs (*nd4*, *nd4l*, *nd5* and *nd1*) were located on the L strand while the other nine were located on the H strand (Table 2). All PCGs started with standard ATN codons for translation initiation (six ATG, three ATT, three ATA, and one ATC). All PCGs ended with the TAA stop codon, whereas in *T. melanocephalum* and *T. sessile*, incomplete stop codons were present (Table S1), as well as in other Dolichoderinae mitogenomes [29,32]. These incomplete stop codons (TA or T) are generated when the coding sequence ends within the 5′-end of the adjacent tRNA. The functional stop codon is generated by the addition of the poly(A) tail in the 3′-end before transcription [33]. 

Mitogenomes have two different non-coding sequences, the control region and the intergenic spacers (IGSs). In *T. ibericum*, in addition to the control region, 24 IGS have been identified (Table 2), the largest one located between the *tRNA-Gln* and *nad2* genes with 104 bp. The rest of IGSs are smaller, ranging from 1 to 76 bp in length. Regarding other ant species, in which their IGSs are described, the total amount of them in *T. ibericum* is the same as in *Solenopsis geminata* [34] but less than in *Atta laevigata* [35] where 30 IGSs were described. However, regarding the total size of all IGSs, in *T. ibericum* it is 719 bp whereas in other species it varies from 519 bp in *S. invicta* to almost 4 kb in *A. laevigata* [35]. 

**Figure 2:**
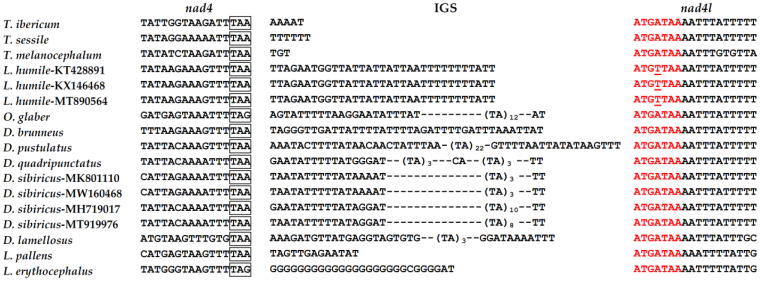
Sequences of the *nad4*/*nad4l* junctions in Dolichoderinae species. In red are the 7 bp conserved regions that forms the overlap between *nad4* and *nad4l* genes in other insect species. The stop codons of the *nad4* gene are in boxes.

Gene overlaps were found at two gene junctions (Table 2). The first one, between *tRNA*-*Ile* and *tRNA*-*Gln* genes, was not present in the other two *Tapinoma* species (Table S1). In *T. sessile*, the spacer between both tRNAs was 89 bp in length and includes a tandem repeat of the TAACTAACT sequence. The second overlap in the *T. ibericum* mitogenome was found between the *atp8* and *atp6* genes. This overlap was conserved in the other Dolichoderinae species. The sequence of this region is ATGATAA, containing the TAA stop codon of the *atp8* gene and the ATG start codon of the *atp6* gene. This same seven-bp sequence forms the overlapping region of *atp8*/*atp6* genes in other insect groups as Lepidoptera [36] or Hemiptera [37]. In fact, the *atp8*/*atp6* gene junction is highly conserved across arthropods [21,38]. It is accepted that insect PCGs are translated from 11 mature transcripts [39]. Two of these mRNAs are bicistronic, one with the *atp8*/*atp6* genes and another with the *nad4l*/*nad4* genes. Conserved overlap between the *nad4l* and *nad4* genes is also a common feature in insect mitogenomes [21]. The conservation of these overlaps has been related with the bicistronic expression of these two gene pairs [39]. However, there is no overlap between the *nad4l* and *nad4* genes in the analyzed Dolichoderinae species (Figure 2). The *nad4* gene starts with the ATGATAA sequence, which is ATGTTAA in *L. humile*, but the TAA of these sequences is not the stop codon of the *nad4l* gene. Mutations upstream of this region generated TAA or TAG stop codons, resulting in an IGS of variable size, from 3 to 88 bp. This IGS is a G run in *E. erythrocephalus*, but in the remaining Dolichoderine species the IGSs are A + T-rich. In *O. glaber* and *Dolichoderus* species, the IGSs showed TA runs with 3 to 22 repeats. The absence of any overlap between the *nad4l* and *nad4* genes was not limited to Dolichoderinae species since it has been observed in species from other ant subfamilies [35,40,41]. In other hymenopteran species, it is also possible to find IGSs in the *atp8*/*atp6* junction. For example, in the wasp *Evania appendigaster*, the *atp6* and *atp8* genes are separated by an IGS of 244 bp [42]. It would be interesting to analyze if the presence of IGSs between the *atp6* and *atp8* or *nad4l* and *nad4* genes affects the transcription and whether the bicistronic transcripts are maintained or not. 

The A + T content of the *T. ibericum* mitogenome was 84.8%. This bias in the nucleotide composition towards A and T nucleotides is a common feature for Hymenoptera mitogenomes [43]. The codon usage also reflects this bias towards A + T codons (Table 3). This bias in the use of codons for the same amino acids can be observed by the relative synonymous codon usage values (RSCU). RSCU is defined as the number of times a codon appears in a gene in relation with the number of expected occurrences under equal codon usage. For all the synonym codons, the RSCU value is higher in NNA or NNT codons (Table 3). The most used codons are A + T-rich: ATT (Ile, 13.26%), TTA (Leu, 11.99%), TTT (Phe, 9.96%) and ATA (Met, 9.39%). Therefore, the four amino acids encoded by these codons are the most abundant in mitochondrial proteins. The A + T bias usage can be also seen in the stop codons. In the *T. ibericum* mitogenome all the used stop codons are TAA (13 times) while the TAG stop codon is not used at all. The TAG stop codon is also not present in the PCGs of the other two *Tapinoma* species (Supplementary Table S1). In fact, most of the PCGs in Dolichoderinae use the TAA stop codon, or incomplete forms of this codon. The only exceptions where the TAG stop codon was present were in the *nad3* and *nad4l* genes in *L. erythrocephalus* and the *nad1* gene in *L. humile*. 

**Figure 3:**
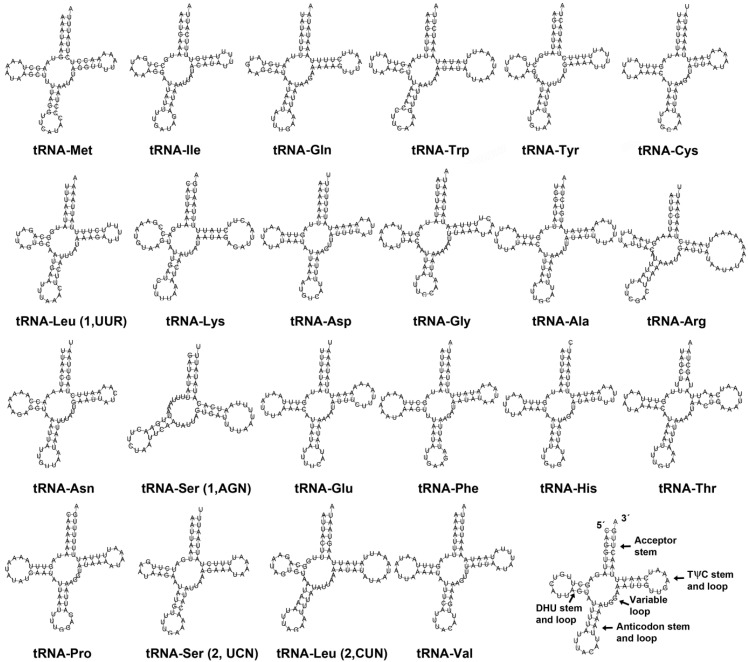
Secondary estructures of all tRNAs.

Figure 3 shows the 22 tRNA genes found in *T. ibericum*. Their lengths range from to 63 bp (*tRNA-Ser1*) to 76 bp (*tRNA-Arg*). These values are close to the length described in other Dolichoderinae species [17,25]. Almost all tRNAs can fold into the typical secondary structure, except for the *tRNA-Ser1* (AGN), which lacks the whole stable sequence in the DHU arm (Figure 3). This feature is common among insects and other metazoans [44]. 

Ribosomal RNA gene annotation is the most difficult step in the mitogenome annotation [21]. The annotations of ribosomal genes were extended until finding adjacent transfer RNAs [33]. The large RNA subunit (*lrRNA*) was located between the *tRNA-Leu* and *tRNA-Val* genes and it has been considered that every base between these two genes was part of the *lrRNA* gene. According this procedure, the 3′ end of the small rRNA (*srRNA*) gene would be delimited by the presence of the *tRNA-Val* gene. However, no tRNA flanks the 5′ end of the *srRNA* gene. Comparison of the annotations of other Dolichoderinae mitogenomes shows great heterogeneity in the placing of the 5′ end of this gene. The analysis of the secondary structure and the presence of conserved motifs at the 5´ end of the *srRNA* gene improves its annotation [21,45]. Since MITOS software takes into account the secondary structure for the annotation [21], the output of this program has been followed for the *srRNA* gene annotation. According our annotation, the lengths of the *lrRNA* and the *srRNA* genes in *T. ibericum* were 1345 and 791 bp respectively, with a total A + T content of 88 and 89%, respectively. 

**Figure 4:**
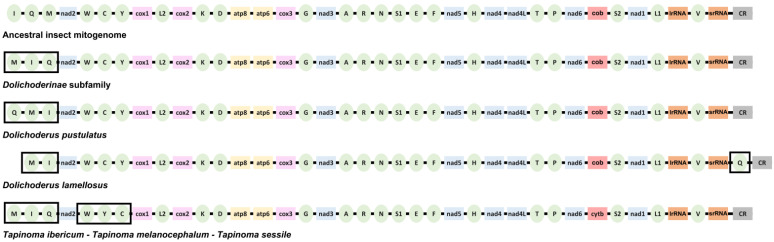
Mitochondrial gene arrangements in Dolichoderinae species.

Gene rearrangements may occur very often in Hymenopteran species [46]. These changes are very valuable and may be used as phylogenetic characters for these insects [47]. In Dolichoderinae, regarding the ancestral pancrustacean–insect mitogenome, four gene rearrangements have been detected (Figure 4). These rearrangements affect the region located between the control region and the *cox1* gene, which includes the *tRNA*-*Ile*, *tRNA-Gln*, *tRNA-Met*, *nad2*, *tRNA-Trp*, *tRNA-Cys* and *tRNA*-*Tyr* genes (IQM-*nad2*-WCY) (Figure 4). The ancestral IQM cluster changes to MIQ in all Dolichoderinae species, except in *D. pustulatus* and *D. lamellosus*. The mitogenome sequences of more than 500 ant species are currently available [24], but the mitogenomes were annotated only in 94 of these species (Supplementary Table S4). The analysis of these species revealed that the MIQ order is the most common in Formicidae, being present in 77 species; among them are most of the Dolichoderinae species as well as species from other ant subfamilies: Formicinae, Myrmicinae, Ponerinae, Dorylinae, Proceratiinae and Pseudomyrmecinae. Therefore, the MIQ order seems to be an ancestral or plesiomorphic character in Formicidae. The QMI cluster of *D. pustulatus* could be the consequence of a later rearrangement, originating from the MIQ ant ancestral cluster. This idea is supported by the presence of the same QMI cluster in four species belonging to other subfamilies: *Cataglyphis aenescens* (Formicinae), *Meranoplus bicolor* (Myrmicinae) and two species from the genus *Ectatoma* (Ectatomminae) (Supplementary Table S2). The ancestral pancrustacean–insect IMQ cluster is present in four species from the Formicinae subfamily: *Nylanderia flavipes* and three species from the genus *Camponotus* (Supplementary Table S4). In these species, a reverse mutation probably generated the IMQ cluster from the MIQ cluster that seems to be the ancestral cluster in ants. In *D. lamellusus*, the *tRNA-Gln* gene is translocated between the *srRNA* gene and the control region (Figure 4). This rearrangement was not present in any other ant species (Supplementary Table S4).

The second region with gene rearrangements in Dolichoderinae species is the WCY cluster (Figure 4). This ancestral gene order is conserved in most of the Formicidae species, being present in 86 out of the 94 annotated mitogenomes (Supplementary Table S2). In Dolichoderinae, changes in the order of these genes have only been detected in *Tapinoma* species, including *T. ibericum*. In the three *Tapinoma* species, this cluster presents a shift between the CY genes and thus a new cluster is formed (WYC) (Figure 4). This change in the cluster could be a synapomorphic character for *Tapinoma* species, and it could be used as a phylogenetic marker in order to distinguish related species belonging to the Dolichoderinae family and more specifically for those ants belonging to the *Tapinoma* genus.

In the dolichoderine *Leptomyrmex pallens*, a translocation of the *tRNA-Asn* gene was described from the ARNSEF cluster to a position located between the *srRNA* gene and the control region [26]. tRNA copies found in the control region could have been originated from duplication events and possibly are non-functional copies [48]. A new analysis using the mitogenome sequence of this species (accession no. KC160533) with the MITOS software has allowed us to determine that it is possible to find the *tRNA-Asn* gene located between the *tRNA-Arg* and the *tRNA-Ser* genes, thus conserving the ARNSEF cluster in all Dolichoderinae species.

Another mitogenome rearrangement detected in several ant species affects the *tRNA-Val* gene, usually located between the *lrRNA* and *srRNA* genes (Figure 4). A translocation of this gene generates the VMIQ cluster. This translocation has been detected in several species belong to the Myrmicinae subfamily [35]. No rearrangements outside the IQM-*nad2*-WCY cluster were observed in Dolichoderinae.

**Figure 5:**
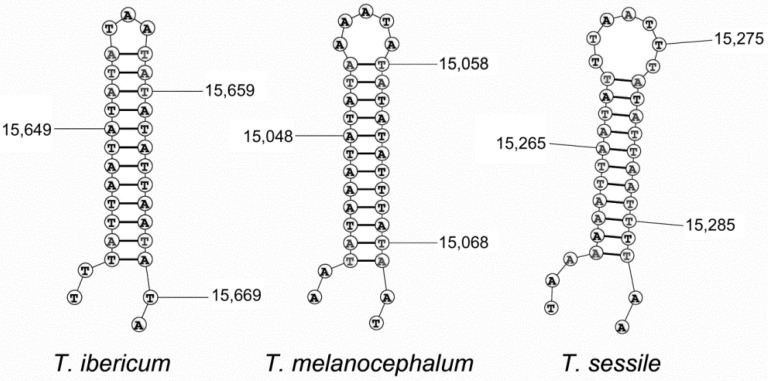
Stem loop structures on last part of the control regions in species from the genus *Tapinoma*. Some nucleotide positions are indicated.

Commonly, the control region is the largest non-coding sequence in mitogenomes. This region is located downstream the *srRNA* gene and has been associated with replication and transcription [49] and is highly variable in base composition and size [50,51]. This region is one of the most difficult regions to retrieve either by PCR or NGS, not only due to the complexity and the sequence composition biased to A + T, but also because of the presence of internal repeats. This region in *T. ibericum* is 341 bp in length, with an A + T richness close to 99% and without any internal repeats. The *T. sessile* control region has no internal repeats and its size is less than 150 bp. However, the control region of *T. melanocephalum* is larger, about 350 bp, and has an internal repeat of 33 bp. In spite of their heterogeneity in size and organization, the control regions of the three *Tapinoma* species contain a sequence of 27–30 bp that could form a stem-loop structure with a perfect match (Figure 5). This structure seems to be conserved in mitogenomes and has been related with the replication mechanism [52]. The existence or not of internal repeats in the control regions seems to be a not conserved feature at generic level. As it happens in *Tapinoma* genus, in other dolichoderine genera it is also possible to find the same variability. So, *D. sibiricus* and *D. quadripunctatus* share an internal repeat of 21 bp, but no internal repeats were present in *D. lamellosus* or *D. pustulatus*. *L. erythrocephalus* shows an internal repeat of 11 bp that was not present in *L. pallens*. *O. glaber* has two different tandem repeats, the first one with a repeat of 21 bp and the second one with 22 bp. Finally, in the control region of *L. humile*, there is an internal repeat of 13 bp, but the number of copies was different in the sequenced mitogenomes, with 5 copies in the North American sample (KT428891) and 18 in the two Asiatic samples (KX146468, MT890564). This difference is the main cause of the different sizes found for the mitogenomes of this species (Table 1).

### 3.2. Phylogenetic Analysis

**Figure 6:**
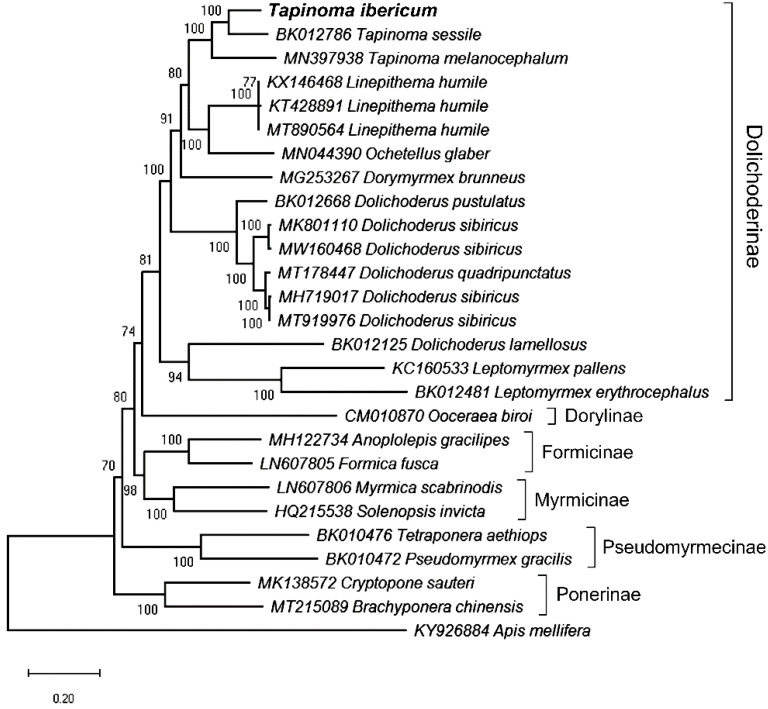
Phylogenetic relationships based on the Maximum Likelihood (ML) analysis. Bootstrap values above 70 are indicated next to the branches. The tree was rooted using *Apis mellifera* as outgroup.

The Maximum Likelihood tree using concatenated PCGs sequences showed Dolichoderinae as being a monophyletic subfamily (Figure 6). *T. ibericum* was clustered together with the other *Tapinoma* species in a well-supported clade. As commented above, the three *Tapinoma* species shared the same gene rearrangement with the WYC array that is absent in the other Dolichoderinae species. These results support the monophyletic nature of the genus. 

As expected, all sequences from *L. humile* are in the same clade. However, it is not the case of the four *D. sibiricus* sequences. Two sequences, one sample from South Korea (MH719017) and other from Russia (MT919976), clustered with *D. quadripunctatus*, whereas the other two *D. sibiricus* sequences are in a different clade. This second clade includes one sequence from South Korea (MK801110) and other from Taiwan (MW160468). Park et al. [15] suggested that these results could indicate the existence of two different species under the taxon *D. sibiricus*. 

The position in the tree of *D. lamellosus* was unexpected. It appears grouped with the *Leptomyrmex* species instead of with the other *Dolichoderus* species. There are no previous data to suggest that the genus *Dolichoderus* could be polyphyletic. On the contrary, previous data support the monophyly of this genus. Ward et al. [53] performed an extensive molecular study using data from 10 nuclear genes in 48 *Dolichoderinae* species, including 6 *Dolichoderus* species, among them *D. lamellosus*. Results showed that *D. lamellosus* together with the other *Dolichoderus* species were clustered in a well-supported clade. Since the sequence of the mitogenome of *D. lamellosus* was directly retrieved from the GenBank it would be desirable to have new sequences of this species to determine the reason for this anomalous position in the phylogenetic tree. 

## 4. Conclusions

In conclusion, the mitogenome sequence of *T. ibericum* provides an important molecular framework for phylogenetic analyses of the family Formicidae. Similar in size to other mitogenomes of the genus *Tapinoma*, the presence of a genus-specific gene rearrangement reinforces the monophyly of the genus. In addition, the phylogenetic tree built with the protein-coding gene sequences of the available dolichoderine mitogenomes and other species of the family Formicidae showed the monophyletic nature of *Tapinoma* species. Moreover, the *T. ibericum* mitogenome description could facilitate the study of the expansion of this potential invasive species in the future.

## Figures and Tables

**Table 1:** The mitochondrial genomes currently sequenced in the subfamily Dolichoderinae.

Species	Genome Size (bp)	Origen Country	Accession Number	Reference
*Dolichoderus lamellosus*	16,234	Costa Rica	BK012125	[24]
*Dolichoderus pustulatus*	16,224	Canada	BK012668	[24]
*Dolichoderus quadripunctatus*	16,017	Poland	MT178447	[15]
*Dolichoderus sibiricus*	16,086	South Korea	MH719017	[25]
	16,044	South Korea	MK801110	[15]
	16,067	Russia	MT919976	unpublished
	16,110	Taiwan	MW160468	unpublished
*Dorymyrmex brunneus*	15,848		MG253267	unpublished
*Leptomyrmex erythrocephalus*	15,546	Australia	BK012481	[24]
*Leptomyrmex pallens*	15,591	New Calcedonia	KC160533	[26]
*Linepithema humile*	16,098	USA	KT428891	[27]
	15,929		KX146468	[20]
	15,934	South Korea	MT890564	[16]
*Ochetellus glaber*	16,259	South Korea	MN044390	[28]
*Tapinoma ibericum*	15,715	Spain	ON746721	This study
*Tapinoma melanocephalum*	15,499	China	MN397938	[29]
*Tapinoma sessile*	15,287	USA	BK012786	[24]

**Table 2:** Annotation of the complete mitogenome of *Tapinoma ibericum*. IGN: intergenic nucleotides. Negative values refer to overlapping nucleotides.

Gene	Anticodon	Strand	Nucleotide Number	Start Codon	Stop Codon	IGN
(*M*) *tRNA-Met*	CAU	H	1–68			3
(*I*) *tRNA-Ile*	GAU	H	72–137			−3
(*Q*) *tRNA-Gln*	UUG	L	135–203			104
*nad2*		H	308–1291	ATA	TAA	1
(W) *tRNA-Trp*	UCA	H	1293–1366			32
(Y) *tRNA-Tyr*	GUA	L	1399–1467			56
(C) *tRNA-Cys*	GCA	L	1501–1567			21
*cox1*		H	1589–3118	ATG	TAA	0
(L1) *tRNA-Leu*	UAA	H	3119–3184			0
*cox2*		H	3185–3874	ATT	TAA	24
(K) *tRNA-Lys*	UUU	H	3899–3971			0
(D) *tRNA-Asp*	GUC	H	3972–4040			69
*atp8*		H	4041–4202	ATC	TAA	−7
*atp6*		H	4196–4864	ATG	TAA	6
*cox3*		H	4871–5653	ATG	TAA	74
(G) *tRNA-Gly*	UCC	H	5728–5795			0
*nad3*		H	5796–6146	ATA	TAA	37
(A) *tRNA-Ala*	UGC	H	6184–6256			22
(R) *tRNA-Arg*	UCG	H	6279–6354			16
(N) *tRNA-Asn*	GUU	H	6371–6439			31
(S1) *tRNA-Ser*	UCU	H	6471–6533			20
(E) *tRNA-Glu*	UUC	H	6554–6624			76
(F) *tRNA-Phe*	GAA	L	6701–6772			0
*nad5*		L	6773–8440	ATA	TAA	0
(H) *tRNA-His*	GUG	L	8441–8509			37
*nad4*		L	8547–9887	ATG	TAA	5
*nad4l*		L	9893–10,180	ATT	TAA	10
(T) *tRNA-Thr*	UGU	H	10,191–10,261			8
(P) *tRNA-Pro*	UGG	L	10,270–10,341			6
*nad6*		H	10,348–10,893	ATG	TAA	23
*cob*		H	10,917–12,038	ATG	TAA	10
(S2) *tRNA-Ser*	UGA	H	12,049–12,115			31
*nad1*		L	12,147–13,094	ATT	TAA	0
(L2) *tRNA-Leu*	UAG	L	13,095–13,166			0
*lrRNA*		L	13,167–14,511			0
(V) *tRNA-Val*	UAC	L	14,512–14,583			0
*srRNA*		L	14,584–15,374			0
Control Region			15,375–15,715			

**Table 3:** Codon usage of *Tapinoma ibericum* mitogenome protein coding genes. A total of 3695 codons were analyzed. RSCU: relative synonymous codon usage. * = termination codon.

Codon	n	%	RSCU	Codon	n	%	RSCU
UUU(F)	368	1.91	9.96	UAU(Y)	198	5.36	1.82
UUC(F)	18	0.09	0.49	UAC(Y)	20	0.54	0.18
UUA(L)	443	5.13	11.99	UAA(*)	13	0.35	2
UUG(L)	13	0.15	0.35	UAG(*)	0	0	0
CUU(L)	31	0.36	0.84	CAU(H)	47	1.27	1.52
CUC(L)	2	0.02	0.05	CAC(H)	15	0.41	0.48
CUA(L)	29	0.34	0.78	CAA(Q)	42	1.14	1.91
CUG(L)	0	0	0	CAG(Q)	2	0.05	0.09
AUU(I)	490	1.91	13.26	AAU(N)	220	5.95	1.86
AUC(I)	22	0.09	0.60	AAC(N)	17	0.46	0.14
AUA(M)	347	1.88	9.39	AAA(K)	112	3.03	1.9
AUG(M)	23	0.12	0.62	AAG(K)	6	0.16	0.1
GUU(V)	63	2.12	1.71	GAU(D)	48	1.30	1.6
GUC(V)	7	0.24	0.19	GAC(D)	12	0.32	0.4
GUA(V)	45	1.51	1.22	GAA(E)	62	1.68	1.8
GUG(V)	4	0.13	0.11	GAG(E)	7	0.19	0.2
UCU(S)	116	3.14	2.75	UGU(C)	31	0.84	1.82
UCC(S)	11	0.30	0.26	UGC(C)	3	0.08	0.18
UCA(S)	98	2.65	2.32	UGA(W)	80	2.17	1.95
UCG(S)	1	0.03	0.02	UGG(W)	2	0.05	0.05
CCU(P)	54	1.46	1.79	CGU(R)	15	0.41	1.4
CCC(P)	12	0.32	0.4	CGC(R)	0	0	0
CCA(P)	51	1.38	1.69	CGA(R)	25	0.68	2.33
CCG(P)	4	0.11	0.13	CGG(R)	3	0.08	0.28
ACU(T)	64	1.73	2.02	AGU(S)	20	0.54	0.47
ACC(T)	4	0.11	0.13	AGC(S)	4	0.11	0.09
ACA(T)	58	1.57	1.83	AGA(S)	75	2.03	1.78
ACG(T)	1	0.03	0.03	AGG(S)	13	0.35	0.31
GCU(A)	50	1.35	2.67	GGU(G)	25	0.68	0.67
GCC(A)	3	0.08	0.16	GGC(G)	8	0.22	0.21
GCA(A)	21	0.57	1.12	GGA(G)	89	2.41	2.39
GCG(A)	1	0.03	0.05	GGG(G)	27	0.73	0.72

## Data Availability

The *T. ibericum* mitogenome sequence was submitted to NCBI (Acc. number ON746721).

## Supplementary Materials

The following are available online at https://www.mdpi.com/article/10.3390/genes13081325/s1, Table S1: Annotation of the mitogenomes of *Tapinoma melanocephalum* and *Tapinoma sessile*; Table S2: Annotation of the mitogenomes of *Dolichoderus lamellosus* and *Dolichoderus pustullatus*; Table S3: Annotation of the mitogenome of *Leptomyrmex erythrocephalus*; Table S4: Gene order in Formicidae regarding the QMI-*nad2*-WCY cluster of the ancestral insect-pancrustaceus mitogenome. References [15,16,20,24,25,26,27,28,29,34,35,40,41,54,55,56,57,58,59,60,61,62,63,64,65,66,67,68,69,70,71,72,73,74,75,76,77] are cited in the supplementary materials.
