# Supplementary material for: Complete Nucleotide Sequence of the Mitogenome of Tapinoma ibericum (Hymenoptera: Formicidae: Dolichoderinae), Gene Organization and Phylogenetics Implications for the Dolichoderinae Subfamily
Source: Genes (Basel). 2022 Jul 25;13(8):1325. doi: 10.3390/genes13081325 (PMC9332376; doi:10.3390/genes13081325)
Supplement: Supplementary file 1 [file genes-13-01325-s001.zip › genes-1795707-supplementary.pdf]

Complete Nucleotide Sequence of the Mitogenome of *Tapinoma ibericum* (Hymenoptera: Formicidae: Dolichoderinae), Gene Organization and Phylogenetics Implications for the Dolichoderinae Subfamily

SUPPLEMENTARY MATERIAL

Table S1. Annotation of the mitogenomes of *Tapinoma melanocephalum* and *Tapinoma sessile.* IGN: intergenic nucleotides. Negative values refer to overlapping nucleotides.

Gene	Anticodon	Strand	Nucleotide number	Start codon	Stop codon	IGN	Nucleotide number	Start codon	Stop codon	IGN
			Tapinoma melanocephalum MN397938 [*]				*Tapinoma sessile* BK012786 [**]			
(M) tRNA-Met	CAU	H	1–68	–	–	6	1-72	–	–	34
(I) tRNA-Ile	GAU	H	75–140	–	–	67	107-173	–	–	1
(Q) tRNA-Gln	UUG	L	208-276	–	–	66	175-243	–	–	89
nad2		H	343-1327	ATA	T--	0	333-1316	ATA	TAA	2
(W) *tRNA-Trp*	UCA	H	1328-1397	–	–	32	1319-1394	–	–	12
(Y) *tRNA-Tyr*	GUA	L	1430-1496	–	–	3	1407-1472	–	–	5
(C) *tRNA-Cys*	GCA	L	1500-1569	–	–	8	1478-1546	–	–	20
cox1		H	1578-3106	ATG	TA-	0	1567-3095	ATG	TA-	0
(L1) *tRNA-Leu*	UAA	H	3107-3172	–	–	0	3096-3162	–	–	0
cox2		H	3173-3862	ATT	TAA	9	3163-3855	ATT	TAA	10
(K) *tRNA-Lys*	UUU	H	3872-3942	–	–	0	3866-3938	–	–	0
(D) *tRNA-Asp*	GUC	H	3943-4010	–	–	0	3939-4005	–	–	0
atp8		H	4011-4172	ATT	TAA	-7	4006-4164	ATA	TAA	-7
atp6		H	4166-4834	ATG	TAA	2	4158-4826	ATG	TAA	3
cox3		H	4837-5619	ATG	TAA	26	4830-5615	ATG	TAA	19
(G) *tRNA-Gly*	UCC	H	5646-5714	–	–	0	5635-5702	–	–	0
nad3		H	5715-6065	ATA	TAA	28	5703-6053	ATT	TAA	30
(A) *tRNA-Ala*	UGC	H	6094-6165	–	–	1	6084-6150	–	–	18
(R) *tRNA-Arg*	UCG	H	6167-6232	–	–	1	6169-6240	–	–	4
(N) *tRNA-Asn*	GUU	H	6234-6301	–	–	15	6245-6312	–	–	40
(S1) *tRNA-Ser*	UCU	H	6317-6377	–	–	7	6353-6413	–	–	12
(E) *tRNA-Glu*	UUC	H	6385-6453	–	–	- 2	6426-6496	–	–	-2
(F) *tRNA-Phe*	GAA	L	6452-6520	–	–	0	6495-6564	–	–	0
nad5		L	6521-8180	ATA	T--	0	6565-8222	ATT	TA-	0
(H) *tRNA-His*	GUG	L	8181-8245	–	–	15	8223-8289	–	–	65
nad4		L	8261-9595	ATG	TAA	3	8355-9683	ATG	TAA	6
nad4l		L	9599-9886	ATT	TAA	1	9690-9977	ATT	TAA	5
(T) *tRNA-Thr*	UGU	H	9888-9952	–	–	3	9983-10046	–	–	12
(P) *tRNA-Pro*	UGG	L	9956-10024	–	–	30	10059-10126	–	–	9
nad6		H	10055-10582	ATT	TAA	10	10136-10681	ATG	TAA	14
cob		H	10593-11717	ATG	TAA	6	10696-11820	ATG	TAA	14
(S2) *tRNA-Ser*	UGA	H	11724-11791	–	–	31	11835-11902	–	–	10
nad1		L	11823-12773	ATT	TAA	0	11913-12860	ATT	TAA	0
(L2) *tRNA-Leu*	UAG	L	12774-12841	–	–	0	12861-12931	–	–	0
lrRNA		L	12842-14187	–	–	0	12932-14299	–	–	0
(V) *tRNA-Val*	UAC	L	14188-14252	–	–	0	14300-13472	–	–	0
srRNA		L	14253-14994	–	–	0	14373-15119	–	–	0
Control Region		-	14995-15499	–	–		15120-15287	–	–	

[*] The annotation of *T. melanocephalum* is basically the performed by Du et al. [24]. We have modified the annotation of the *nad2* gene to avoid overlapping with the *tRNA-Trp*.

[**] The *T. sessile* mitogenome was not annotated by the authors [24]. For this we have reordered the sequence to begin with the *tRNA-Met* gene and then annotated the mitogenome using the same procedure as for *T. ibericum*.

Table S2. Annotation of the mitogenomes of *Dolichoderus lamellosus* and *Dolichoderus pustullatus.* IGN: intergenic nucleotides. Negative values refer to overlapping nucleotides.

Gene	Anticodon	Strand	Nucleotide number	Start codon	Stop codon	IGN	Nucleotide number	Start codon	Stop codon	IGN
			Dolichoderus lamellosus **BK012125** *				***Dolichoderus pustulatus*** **BK012668** *			
(M) tRNA-Met	CAU	H	1-72	–	–	9	1-67	–	–	1
(I) tRNA-Ile	GAU	H	82-153	–	–	147	69-134	–	–	158
(Q) tRNA-Gln			**translocated**							
nad2		H	301-1303	ATA	TAA	107	293-1270	ATA	TAA	2
(W) *tRNA-Trp*	UCA	H	1411-1481	–	–	13	1273-1338	–	–	5
(C) *tRNA-Cys*	GCA	L	1495-1564	–	–	49	1344-1414	–	–	15
(Y) *tRNA-Tyr*	GUA	L	1614-1684	–	–	42	1430-1495	–	–	34
cox1		H	1727-3254	ATG	T--	0	1530-3057	ATG	T--	0
(L1) *tRNA-Leu*	UAA	H	3255-3322	–	–	0	3058-3121	–	–	0
cox2		H	3323-4003	ATT	TAA	92	3122-3811	ATT	TAA	20
(K) *tRNA-Lys*	UUU	H	4096-4167	–	–	34	3832-3903	–	–	5
(D) *tRNA-Asp*	GUC	H	4202-4273	–	–	0	3909-3979	–	–	0
atp8		H	4274-4441	ATT	TAA	-5	3980-4135	ATT	TAA	-5
atp6		H	4435-5105	ATG	TAA	41	4129-4797	ATG	TAG	9
cox3		H	5147-5941	ATG	TAA	8	4807-5595	ATG	TAA	41
(G) *tRNA-Gly*	UCC	H	5950-6017	–	–	0	5637-5695	–	–	20
nad3		H	6018-6368	ATA	TAA	22	5716-6063	ATA	TAA	44
(A) *tRNA-Ala*	UGC	H	6391-6455	–	–	24	6108-6178	–	–	31
(R) *tRNA-Arg*	UCG	H	6480-6546	–	–	84	6210-6276	–	–	15
(N) *tRNA-Asn*	GUU	H	6631-6702	–	–	30	6292-6357	–	–	35
(S1) *tRNA-Ser*	UCU	H	6733-6792	–	–	39	6393-6455	–	–	7
(E) *tRNA-Glu*	UUC	H	6832-6907	–	–	1	6463-6537	–	–	5
(F) *tRNA-Phe*	GAA	L	6909-6978	–	–	6	6543-6612	–	–	0
nad5		L	6985-8658	ATA	TAA	12	6613-8275	ATT	T--	9
(H) *tRNA-His*	GUG	L	8671-8739	–	–	42	8285-8357	–	–	25
nad4		L	8782-10134	ATG	TAA	39	8583-9923	ATG	TAA	88
nad4l		L	10174-10446	ATT	TAA	30	10012-10281	ATT	TAA	33
(T) *tRNA-Thr*	UGU	H	10477-10547	–	–	7	10315-10386	–	–	7
(P) *tRNA-Pro*	UGG	L	10555-10628	–	–	53	10394-10462	–	–	36
nad6		H	10682-11215	ATC	TAA	56	10499-11017	ATC	TAA	10
cob		H	11272-12438	ATG	TAA	40	11028-12155	ATG	TAA	29
(S2) *tRNA-Ser*	UGA	H	12479-12548	–	–	14	12285-12352	–	–	9
nad1		L	12563-13519	ATT	TAG	0	12362-13309	ATA	TAA	3
(L2) *tRNA-Leu*	UAG	L	13520-13586	–	–	0	13313-13380	–	–	0
lrRNA		L	13587-14124	–	–	800	13381-14725	–	–	0
(V) *tRNA-Val*	UAC	L	14925-14990	–	–	0	14726-14798	–	–	0
srRNA		L	14991-15785	–	–	42	14799-15577	–	–	0
(Q) tRNA-Gln	UUG	L	15828-15896	–	–	0		–	–	
Control Region			15897-16234				15578-16142			0
(Q) tRNA-Gln	UUG	L					16143-16211			0
IGS							16212-16224			

* The *D. lamellosus* and *D. pustulatus* mitogenomes were not annotated by the authors [24]. To do this we have reordered the sequences to begin with the *tRNA-Met* gene and then annotated the mitogenomes using the same procedure as for *T. ibericum*.

Table S3. Annotation of the mitogenome of *Leptomyrmex erythrocephalus*. IGN: intergenic nucleotides. Negative values refer to overlapping nucleotides.

Gene	Anticodon	Strand	Nucleotide number	Start codon	Stop codon	IGN
			Leptomyrmex erythrocephalus **BK012481** *			
(M) tRNA-Met	CAU	H	1-69	–	–	0
(I) tRNA-Ile	GAU	H	70-135	–	–	16
(Q) tRNA-Gln	UUG	L	152-221	–	–	73
nad2		H	295-1278	ATT	TAA	33
(W) *tRNA-Trp*	UCA	H	1312-1380	–	–	0
(C) *tRNA-Cys*	GCA	L	1380-1446	–	–	22
(Y) *tRNA-Tyr*	GUA	L	1469-1535	–	–	22
cox1		H	1558-3083	ATG	T--	0
(L1) *tRNA-Leu*	UAA	H	3084-3151	–	–	0
cox2		H	3152-3832	ATA	TAA	22
(K) *tRNA-Lys*	UUU	H	3854-3925	–	–	-1
(D) *tRNA-Asp*	GUC	H	3925-3997	–	–	0
atp8		H	3998-4153	ATC	TAG	-4
atp6		H	4150-4815	ATA	TAA	6
cox3		H	4822-5622	ATG	TAA	12
(G) *tRNA-Gly*	UCC	H	5635-5700	–	–	0
nad3		H	5701-6051	ATC	TAG	7
(A) *tRNA-Ala*	UGC	H	6059-6123	–	–	6
(R) *tRNA-Arg*	UCG	H	6130-6198	–	–	14
(N) *tRNA-Asn*	GUU	H	6213-6277	–	–	2
(S1) *tRNA-Ser*	UCU	H	6280-6339	–	–	3
(E) *tRNA-Glu*	UUC	H	6343-6411	–	–	-2
(F) *tRNA-Phe*	GAA	L	6410-6474	–	–	0
nad5		L	6475-8143	ATA	T--	0
(H) *tRNA-His*	GUG	L	8144-8214	–	–	9
nad4		L	8224-9546	ATG	TAA	27
nad4l		L	9574-9861	ATT	TAG	1
(T) *tRNA-Thr*	UGU	H	9863-9928	–	–	11
(P) *tRNA-Pro*	UGG	L	9940-10012	–	–	16
nad6		H	10029-10568	ATG	TAA	2
cob		H	10571-11678	ATG	T--	0
(S2) *tRNA-Ser*	UGA	H	11679-11744	–	–	4
nad1		L	11749-12698	ATG	TA-	3
(L2) *tRNA-Leu*	UAG	L	12702-12770	–	–	0
lrRNA		L	12771-14115	–	–	0
(V) *tRNA-Val*	UAC	L	14116-14177	–	–	0
srRNA		L	14178-14995	–	–	0
Control Region			14996-15546			

* The *Leptomyrmex erythrocephalus* mitogenome was not annotated by the authors [24]. To perform the annotation we have reordered the sequences to begin with the *tRNA-Met* gene and then annotated the mitogenome using the same procedure as for *T. ibericum*.

Table S4. Gene order in Formicidae regarding the QMI-*nad2*-WCY cluster of the ancestral insect-pancrustaceus mitogenome. Dolichoderinae species are shown shaded.

Gene order	Species	Subfamily	Accesion number	Reference
**MIQ *nad2* WCY**	*Dolichoderus quadripunctatus*	Dolichoderinae	MT178447	[15]
	Dolichoderus sibiricus	Dolichoderinae	MH719017	[25]
			MK801110	[15]
			MT919976	Unpublished
			MW160468	Unpublished
	Dorymyrmex brunneus	Dolichoderinae	MG253267	Unpublished
	Leptomyrmex erythocephalus *	Dolichoderinae	BK012481	[24]
	Leptomyrmex pallens	Dolichoderinae	KC160533	[26]
	Linepithema humile	Dolichoderinae	KT428891	[27]
			KX146468	[20]
			MT890564	[16]
	Ochetellus glaber	Dolichoderinae	MN044390	[28]
	Acropyga donisthorpei	Formicinae	MH158404	Unpublished
	Acropyga fuhrmanni	Formicinae	MH158405	Unpublished
	Acropyga goeldii	Formicinae	MH158403	Unpublished
	Acropyga guianensis	Formicinae	MH158406	Unpublished
	Acropyga kinomurai	Formicinae	MH158407	Unpublished
	Acropyga myops	Formicinae	MH158408	Unpublished
	Acropyga pallida	Formicinae	MH158409	Unpublished
	Acropyga panamensis	Formicinae	MH158410	Unpublished
	Acropyga sauteri	Formicinae	MH158411	Unpublished
	Acropyga smithii	Formicinae	MH158413	Unpublished
	Anoplolepis gracilipes	Formicinae	MH122734	[54]
	Colobopsis nipponica	Formicinae	MW067133	[55]
	Formica fusca	Formicinae	LN607805	[56]
	Formica selysi	Formicinae	KP670862	[40]
	Formica sinae	Formicinae	OL581666	Unpublished
	Formica sp.	Formicinae	MT941916	Unpublished
	Formica rufa	Formicinae	MT862420	Unpublished
	Lasius niger	Formicinae	MT862424	Unpublished
	Lasius spathepus	Formicinae	MW074965	[57]
	Lepisiota frauenfeldi	Formicinae	OK569858	Unpublished
	Polyrhachis dives	Formicinae	KT266831	[58]
	Aphaenogaster famelica	Myrmicinae	MK801109	[59]
	Aphaenogaster japonica	Myrmicinae	MW915466	Unpublished
	Atta cephalotes	Myrmicinae	HQ415764	[60]
	Atta colombica	Myrmicinae	KY950644	[61]
	Atta laevigata	Myrmicinae	KC346251	[35]
	Atta opaciceps	Myrmicinae	KY950643	[61]
	Atta sexdens	Myrmicinae	MF591717	[61]
	Atta texana	Myrmicinae	MF417380	[61]
	Cardiocondyla obscurior	Myrmicinae	KX951753	[62]
	Carebara diversa	Myrmicinae	OL689134	Unpublished
	Crematogaster matsumurai	Myrmicinae	OM328370	Unpublished
	Crematogaster teranishii	Myrmicinae	MK940828	[63]
	Messor structor	Myrmicinae	OL581665	[64]
	Monomorium pharaonis	Myrmicinae	MT038041	Unpublished
	Monomorium triviale	Myrmicinae	LC605004	[65]
	Myrmica scabrinodis	Myrmicinae	LN607806	[56]

	Octostruma stenognatha	Myrmicinae	KX758608	[66]
	Pheidole nodus	Myrmicinae	MW429351	[67]
	Pristomyrmex punctatus	Myrmicinae	AB556946	[68]
	Solenopsis geminata	Myrmicinae	HQ215537	[34]
	Solenopsis invicta	Myrmicinae	HQ215538	[34]
	Solenopsis richteri	Myrmicinae	HQ215539	[34]
	Stenamma expolitum	Myrmicinae	MT357020	Unpublished
	Stenamma megamanni	Myrmicinae	MT357022	Unpublished
	Stenamma muralla	Myrmicinae	MT357023	Unpublished
	Tetramorium caespitum	Myrmicinae	MT862406	Unpublished
	Tetramorium tsushimae	Myrmicinae	MW429350	[69]
	Wasmannia auropunctata	Myrmicinae	KX146469	[20]
	Vollenhovia emeryi	Myrmicinae	KU550061	[70]
	Ooceraea biroi	Dorylinae	CM010870	[71]
	Cryptopone sauteri	Ponerinae	MK138572	[72]
	Ectomomyrmex javanus	Ponerinae	MK496222	[73]
	Hypoponera sauteri	Ponerinae	MT215090	Unpublished
	Proceratium itoi	Proceratiinae	MT215091	Unpublished
	Pseudomyrmex concolor	Pseudomyrmecinae	BK010475	[74]
	Pseudomyrmex dendroicus	Pseudomyrmecinae	BK010473	[74]
	Pseudomyrmex pallidus	Pseudomyrmecinae	BK010383	[74]
	Pseudomyrmex elongatus	Pseudomyrmecinae	BK010474	[74]
	Pseudomyrmex gracilis	Pseudomyrmecinae	BK010472	[74]
	Pseudomyrmex feralis	Pseudomyrmecinae	BK010379	[74]
	Pseudomyrmex ferrugineus	Pseudomyrmecinae	BK010380	[74]
	Pseudomyrmex flavicornis	Pseudomyrmecinae	BK010381	[74]
	Pseudomyrmex janzeni	Pseudomyrmecinae	BK010382	[74]
	Pseudomyrmex particeps	Pseudomyrmecinae	BK010384	[74]
	Pseudomyrmex peperi	Pseudomyrmecinae	BK010385	[74]
	Pseudomyrmex veneficus	Pseudomyrmecinae	BK010386	[74]
	Tetraponera aethiops	Pseudomyrmecinae	BK010476	[74]
	Tetraponera rufonigra	Pseudomyrmecinae	BK010387	[74]
**MIQ *nad2* WYC**	*Tapinoma ibericum**	Dolichoderinae	ON746721	This study
	Tapinoma melanocephalum	Dolichoderinae	MN397938	[29]
	Tapinoma sessile *	Dolichoderinae	BK012786	[24]
**QMI *nad2* WCY**	*Dolichoderus pustulatus**	Dolichoderinae	BK012668	[24]
	Cataglyphis aenescens	Formicinae	OL581664	Unpublished
	Meranoplus bicolor	Myrmicinae	OK183534	Unpublished
	Ectatoma ruidum	Ectatomminae	MG870209	[41]
	Ectatoma tuberculatum	Ectatomminae	MG870208	[41]
**Q control-region MI *nad2* WCY**	*Dolichoderus lamellusus* *	Dolichoderinae	BK012125	[24]
**IQM *nad2* WCY**	*Camponotus concavus*	Formicinae	MK225553	[75]
	Camponotus atrox	Formicinae	KT159775	[76]
	Camponotus japonicus	Formicinae	OK509076	Unpublished
	Nylanderia flavipes	Formicinae	MN654113	[77]
**M *nad2* NWIQCY**	*Stenamma diecki*	Myrmicinae	MT357019	Unpublished
	Stenamma impar	Myrmicinae	MT357021	Unpublished
**QCIM *nad2* WY**	*Brachyponera chinensis*	Ponerinae	MT215089	Unpublished
**CIQMT *nad2* W**	*Stigmatomma silvestrii*	Amblyoponinae	MT215092	Unpublished

* The mitogenome of these species have been annotated in this study
